# Expression of DNA mismatch repair proteins in melanoma patients treated with immune checkpoint inhibitors

**DOI:** 10.1007/s00432-022-04002-4

**Published:** 2022-04-13

**Authors:** T. Gambichler, C. Finis, N. Abu Rached, C. H. Scheel, J. C. Becker, K. Lang, H. U. Käfferlein, T. Brüning, N. Abolmaali, L. Susok

**Affiliations:** 1 Skin Cancer Center, Department of Dermatology, Ruhr-University Bochum, Gudrunstraße, 5644791 Bochum, Germany; 2 Translational Skin Cancer Research, DKTK Partner Site Essen/Düsseldorf, West German Cancer Center, Dermatology, University Duisburg-Essen, Essen, Germany; 3 German Cancer Research Center (DKFZ), Heidelberg, Germany; 4 Institute for Prevention and Occupational Medicine of the German Social Accident Insurances, Ruhr-University Bochum (IPA), Bochum, Germany; 5 Institute for Diagnostic and Interventional Radiology and Nuclear Medicine, St. Josef Hospital Bochum, Ruhr University Bochum, Bochum, Germany

**Keywords:** Cutaneous melanoma, Immune checkpoint inhibitors, Ipilimumab, Pembrolizumab, Nivolumab, DNA mismatch repair proteins, Mismatch repair deficiency, Microsatellite instability

## Abstract

**Purpose:**

To investigate the protein expression of DNA mismatch repair (MMR) proteins in patients with cutaneous melanoma (CM) under immune checkpoint inhibitor (ICI) therapy.

**Methods:**

Immunohistochemistry was performed on tumor tissue for MMR proteins MLH1, MSH2, MSH6, and PMS2 in 50 metastatic CM patients treated with ICI (ipilimumab, nivolumab, pembrolizumab).

**Results:**

Best overall response (BOR) rate was 48% (24/50). Reduced MMR protein expression (nuclear expression in < 80% of tumor cells) was observed in 8 patients (16%). Compared to other clinical parameters, baseline neutrophil/lymphocyte ratio and reduced intratumoral MMR protein expression (*P* = 0.0033) were determined as the only parameters significantly associated with favorable BOR. However, in this small study population, reduced MMR protein expression did not reach statistical significance in multivariate analysis.

**Conclusion:**

Reduced MMR protein expression is observed in CM and might predict favorable BOR in patients treated with ICI, as was observed for other entities. However, these findings need to be substantiated in larger patient cohorts.

## Introduction

The incidence of cutaneous melanoma (CM) is increasing worldwide, with more than 55,000 deaths per year attributed to this malignancy. Immune checkpoint inhibitors (ICI), specifically antibodies against programmed death protein 1 (PD-1, pembrolizumab, nivolumab) and cytotoxic T lymphocyte associated protein 4 (CTLA-4, ipilimumab) have become first-line treatment regimens for patients with advanced CM. However, approximately 50% of patients do not respond to ICI and it is still difficult to predict who will or will not respond. Hence, there is an urgent need for biomarkers predicting treatment outcome to ICI, particularly considering ICI-mediated adverse events and high costs (Whiteman et al. 2016; Bai et al. 2021; Schadendorf et al. 2018). For many other malignancies, such as colorectal, endometrial, prostate, and bladder cancers, deficiencies in the DNA mismatch repair (MMR) machinery, evident in microsatellite instability (MSI), carry prognostic significance. For example, the prognosis of most cancers with high-level MSI resulting from MMR deficiency is comparatively favorable and indicates responsiveness to treatment with ICI (Breakstone 2021; Germano et al. 2021; Mandal et al. 2019; Cho et al. 2021). At least in part, this is thought to be due to an increased number of frameshift mutations in these tumors resulting in the presentation of neoantigens. In the present study, we aimed to determine protein expression of MMR components in patients with unresectable or metastatic CM and to evaluate whether protein expression is associated with response to ICI treatment.

## Methods

### Patients

We included patients with inoperable stage III and IV CM who received treatment with ICI. Therapy and staging procedures were performed in accordance with national guidelines for the management of CM and interdisciplinary tumor board decisions (Schadendorf et al. 2018). ICI, including mono-nivolumab, mono-pembrolizumab, mono-ipilimumab, and ipilimumab plus nivolumab were administered according to label (Marconcini et al. 2018). Complete staging was performed including lymph node ultrasound, thoracic and/or abdominal computed tomography or positron emission tomography in combination with computer tomography, and cranial magnetic resonance tomography (Schadendorf et al. 2018). The criteria for best overall response (BOR) were used in accordance with RECIST 1.1 (Eisenhauer et al. 2009). Laboratory parameters assessed at the beginning of ICI treatment included full blood count (neutrophil/lymphocyte ratio), BRAF status, lactate dehydrogenase (LDH) levels, and serum S100B.

### Immunohistochemistry

Immunohistology for MMR proteins was performed as previously described (Gambichler et al. 2021a, b). We predominantly used tissue of the primary tumor or, alternatively, lymph node and cutaneous metastases. For immunostaining, we used rabbit monoclonal antibodies against PMS2 and MSH6 (M3646; Agilent DAKO, Hamburg, Germany), and mouse monoclonal antibodies against MLH1 (M3640; Agilent DAKO, Hamburg, Germany) and MSH2 (M3639; Agilent DAKO, Hamburg, Germany) (Gambichler et al. 2021a, b).

### Microscopic evaluation

Microscopic evaluation was carried out using slides scanned at 20-fold magnification with the Nanozoomer Whole Slide Scanner (Hamamatsu, Herrsching am Ammersee, Germany). All scans were assessed by means of the NDP.view2 software (Hamamatsu Photonics, Hamamatsu City, Japan). Microscopic evaluation was performed as previously described (Gambichler et al. 2021a, b). Briefly, nuclear staining of each MMR protein was evaluated in all tumor cells in 5–10 field of views. Protein expression was expressed as the percentage of nuclear-stained tumor cells relative to all tumor cells assessed. Per definition, MMR deficiency was declared when there was complete absence of nuclear staining for at least one protein (Umar et al. 2004; Hashmi et al. 2017*).* Furthermore, cases with an MMR protein expression of less than 80% were classified as diminished MMR protein expression status.

### Statistics

Data analysis was performed using the statistical package MedCalc Software version 20.014 (MedCalc Software, Ostend, Belgium). Where appropriate, univariate analysis was performed using the ROC curve analysis of continuous variables (e.g., NRL), Kaplan–Meier statistics, Spearman correlation procedure, and *χ*^2^ test. Data analysed with a *P* value < 0.1 was included in the multivariate analysis. The latter was carried-out by means of logistic regression. *P* values < 0.05 were considered significant.

## Results

The study population consisted of 50 patients with CM, including 19 women (38%) and 31 men (62%) with a median age of 66.1 (SD ± 11.7). According to the AJCC 8th edition, 9 patients (18%) were unresectable stage III and 41 patients (82%) were stage IV prior to the start of ICI treatment (Table 1). ICI was initiated with anti-PD1 agents in 33 patients (66%), ipilimumab monotherapy in 9 patients (18%), and nivolumab plus ipilimumab in 8 patients (16%). Over the whole treatment history, 48 patients (96%) received anti-PD1 agents, 19 patients (38%) received ipilimumab monotherapy, and 17 patients (34%) nivolumab plus ipilimumab during the total follow-up resulting in a median of 13.1 ICI cycles (range 1–64 cycles). BOR rate according to RECIST 1.1 was 48% (24/50). The median progression-free survival (PFS) was 5.5 months (range 3–68 months). A median 5-year CM-specific survival (CMSS) of 21.5 months (range 3–76 months) was observed, corresponding to 21 CM-specific deaths (58%). Immune-related adverse events of any grade were reported in 24 patients (48%).

**Table 1:** Clinical characteristics and results of DNA mismatch repair protein analysis in patients (*n* = 50) with advanced cutaneous melanoma (CM) treated with immune checkpoint inhibitors

Parameters	Data
Age at diagnosis*	66.1 (SD ± 11.7) years
Sex	
M/F	31/19 (62%/38%)
Tumor stage at diagnosis (AJCC 2018)	
IIIC	6 (12%)
IIID	3 (6%)
IV (M1a)	6 (12%)
IV (M1b)	12 (24%)
IV (M1c)	22 (44%)
IV (M1d)	1 (2%)
Mismatch repair protein expression* (% positive cells)	
MLH1	95.7% (47.9–99.7)
Cases with reduced expression (< 80%)	2 (4%)
MSH2	95.4% (7.1–99.2)
Cases with reduced expression (< 80%)	5 (10%)
MSH6	97% (80.5–99.8)
Cases with reduced expression (< 80%)	0 (0%)
PMS2	95.8% (66.5–99)
Cases with reduced expression (< 80%)	1 (2%)
Outcome	
CM progress (no/yes)	12/38 (24%/76%)
Median progression-free survival time	5.5 months(3–68)
CM (survived/deceased)	21/29 (42%/58%)
CM-specific survival time	21.5 months (3/76)

*Medians and range

**Fig. 1:**
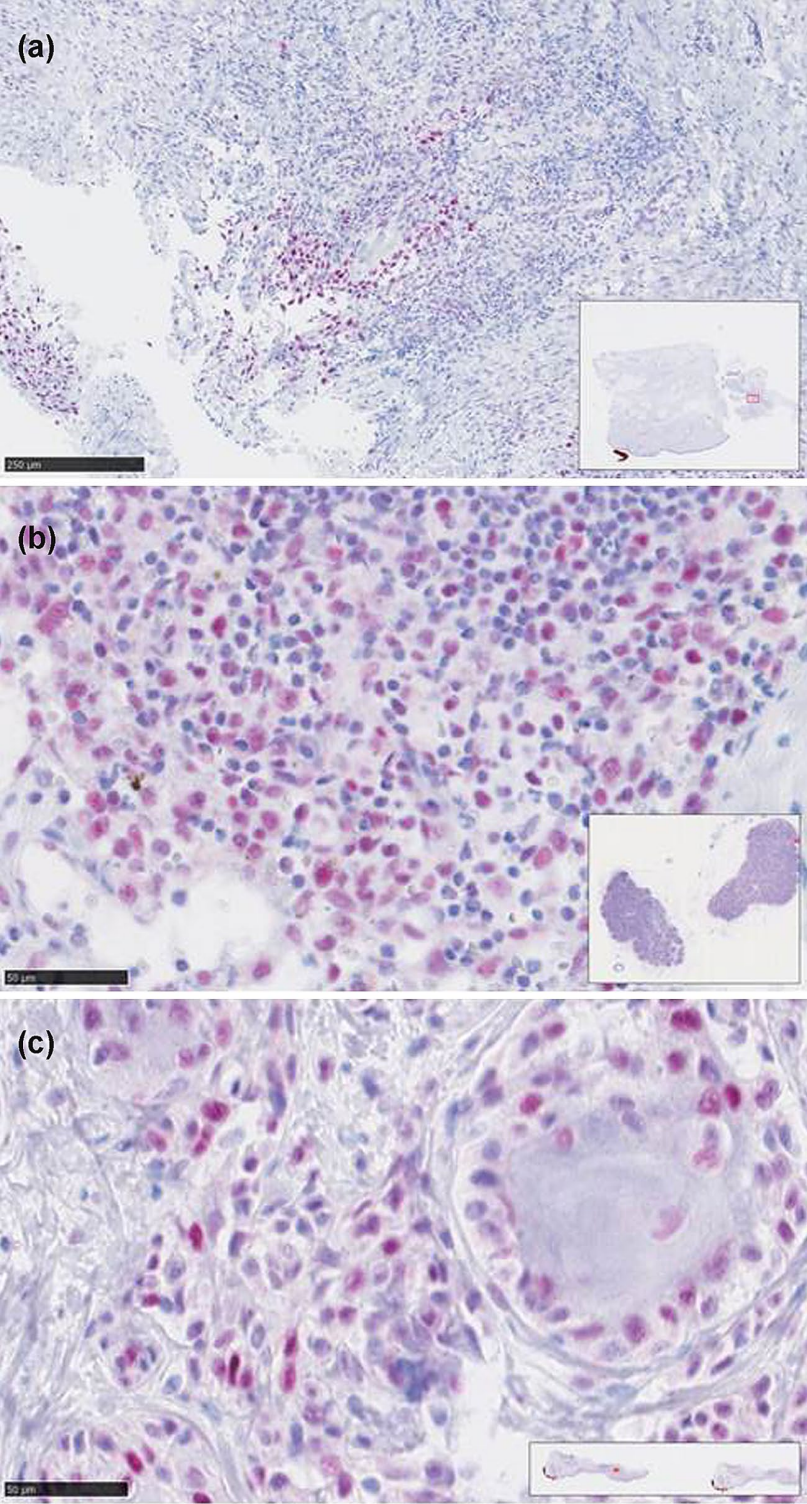
The image demonstrates reduced (< 80% nuclear immunoreactivity) mismatch repair protein (**a**, MSH2; **b**, MLH1; **c**, PMS2) expression in 3 patients with metastatic cutaneous melanoma who responded to immune checkpoint inhibitor treatment

**Fig. 2:**
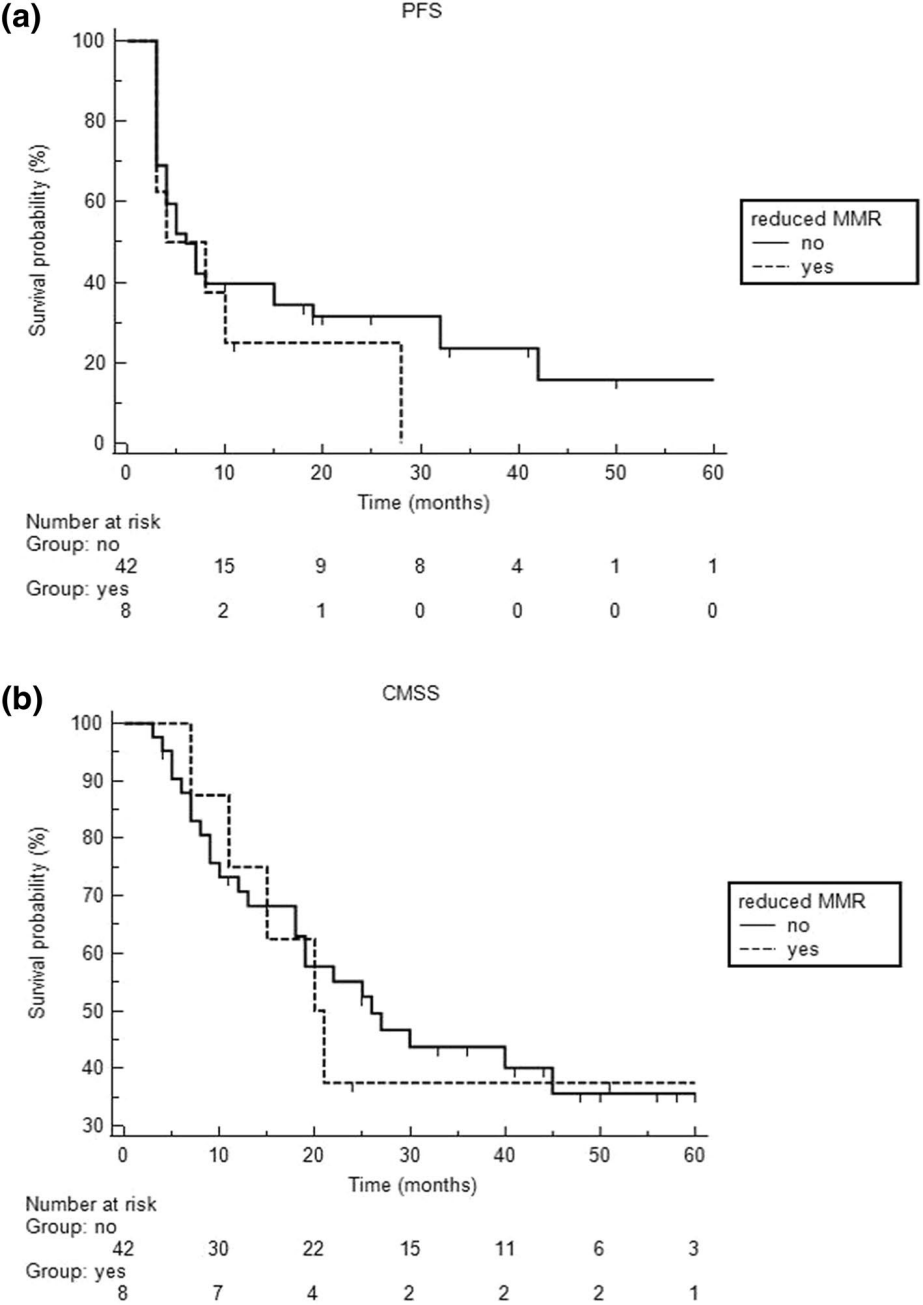
The figure shows the Kaplan–Meier curves of the progression-free survival (PFS, **a**) and the cutaneous melanoma-specific survival (CMSS, **b**) of immunotherapy-treated patients with at least one reduced mismatch repair (MMR) protein on immunohistochemistry. Compared to patients without reduced MMR expression those with reduced MMR expression did not significantly differ with respect to progression-free survival [*P* = 0.35 (hazard ratio 0.61, 95% CI 0.22–1.72)] and the cutaneous melanoma-specific survival [*P* = 0.86 (hazard ratio 0.92, 95% CI 0.34–2.49)]

Taken together, nuclear staining of all four MMR proteins assessed (PMS2, MSH6, MLH1 and MSH2) was very high with median nuclear expression levels of over 95%. Consequently, expression of each MMR protein was strongly correlated with expression of the other ones (*P* < 0.001). Thus, none of the patients showed MMR-deficiency as defined by complete loss of nuclear expression. However, reduced MMR protein expression as defined by nuclear expression in less than 80% of tumor cells was observed in 8 patients (16%), of which 5 showed diminished MSH2 expression, 3 MLH1, and 1 reduced PMS2 expression (Fig. 1). In contrast to gender, age, S100B, LDH, BRAF-status, high-tumor thickness, and stage of disease, low neutrophil/lymphocyte ratio at baseline [median 3.1 (1–9.7), associated criterion ≤ 3.1, AUC 0.70)] and reduced MMR protein expression were determined as the only parameters significantly associated with BOR (*P* = 0.0092 and *P* = 0.0033, respectively). However, the latter two parameters did not significantly correlate with progression-free survival and CMSS (Fig. 2). Using multivariate logistic regression analysis, we found that low neutrophil/lymphocyte ratio remained in the model as significant predictor for BOR, whereas reduced MMR protein expression did not reach statistical significance (*P* = 0.0023 vs. *P* = 0.061, respectively).

## Discussion

The American Society for Clinical Pathology and similar authorities strongly recommend the assessment of MSI/MMR biomarkers in colorectal cancer for better prognostic stratification of patients. This recommendation is emphasized by recent evidence of MSI as a positive predictive factor for response to ICI (Vatrano et al. 2020). Hence, the Food and Drug Administration has recently approved pembrolizumab as first-line therapy for MSI-high/deficient MMR metastatic colorectal cancer (Zhu et al. 2021). MSI-high/deficient MMR status is observed most frequently in colorectal (up to 15%) and gastric cancer (about 10%) (Zhu et al. 2021), and less frequently in hepatocellular carcinoma and esophageal and pancreatic adenocarcinoma (< 5%) (Zhu et al. 2021). Because of the dramatic response of MSI-high/deficient MMR tumors to ICI, MSI/MMR testing has increased significantly in many solid malignancies.

By contrast, data on MSI/MMR in cutaneous malignancies are relatively scarce. In non-melanoma skin cancer, complete loss of MMR protein expression has been rarely observed, whereas decreased expression similar to the data presented here has been observed in cutaneous squamous cell carcinoma and Merkel cell carcinoma (Gambichler et al. 2021a, b). Moreover, MSI-high/deficient MMR status might also be relevant in CM and its responsiveness to ICI (Buder-Bakhaya and Hassel 2018; Kubeček and Kopecký 2016). Korabiowska et al. (2004) studied MMR proteins both by immunohistochemistry and in situ hybridization in 59 CMs. In line with our data, they proposed that in CM, a decreased expression of MMR proteins, rather than a total loss, might be prognostic significance (Korabiowska et al. 2004). Alvino et al. (2014) also reported a reduction of MLH1, MSH2, and PMS2 protein expression in CM compared to melanocytic nevi. Notably, high MSH6 expression in CM was significantly associated with an increased risk of CM mortality, a finding not corroborated by our study. Roncati (2018) reported a patient with mucosal melanoma and MMR-deficiency for MSH6 who experienced long-term disease control under PD-1 blockade with pembrolizumab. Moreover, Ponti et al. (2020) studied 14 CM patients receiving ICI. They performed immunohistochemistry for MLH1, MSH2, MSH6, and PMS2 on primary tumors and several metastases. Ponti et al. (2020) observed MSH6 deficiency in 3 tumor samples (primary melanoma and two metastases) of one patient who had the most successful response to ICI. Taken together, anecdotal evidence in patients with melanoma suggests that in the relatively rare event where it occurs, MMR deficiency positively predicts response to ICI.

Wang et al. (2022) recently described a novel computational tool (CODEFACS) and a supporting immune interactions framework (LIRICS), enabling an averaged “virtual single-cell” characterization of the tumor microenvironment from bulk tumor expression data. They detected a shared repertoire of cell-type-specific ligand-receptor interactions unique to the tumor microenvironment of MMR-deficient malignancies. Wang et al. (2022) focused on CM, which is known for one of the best response rates to ICI, and where a wealth of publicly available bulk expression datasets exists of patients receiving ICI. Using machine learning techniques, they detected a subset of intercellular tumor microenvironment interactions that stratified survival outcomes of CM patients receiving ICI therapy better than some recently published transcriptomics-based methods. Thus, hypermutated CM with deficient MMR showed the best response to ICI treatment (Wang et al. 2022).

The main limitations of the present study include the small sample size and absence of testing for microsatellite instability using multiplex PCR. The data presented here support other findings that MMR deficiency, defined by complete loss of nuclear expression in tumor cells, appears to be a rare event, since it was not observed in this cohort of 50 CM patients. Similar to Merkel cell carcinoma (Gambichler et al. 2021b), however, we detected a decrease of single MMR proteins in 16% of cases, whereas diminished MMR expression was most frequently observed for MSH2. Importantly, all patients with reduced MMR protein expression responded to ICI. Specifically, decreased MMR protein expression was significantly associated with BOR rates in univariate analysis. In multivariate analysis, however, this parameter did not reach statistical significance, which was very likely due to the limited number of patients investigated. Thus, in contrast to Wang et al. (2022), we did not observe a correlation between MMR expression status and PFS or CMSS. In conclusion, reduced MMR protein expression is not uncommon in CM and might be a predictor for improved response to treatment with ICI. However, the present data have to be substantiated in larger trials.

## Data Availability

Derived data supporting the findings of this study are available from the corresponding author on reasonable request.
